# Editors’ Comments on the Special Issue “Social Determinants of Mental Health”

**DOI:** 10.3390/ijerph18083957

**Published:** 2021-04-09

**Authors:** Emma Motrico, Jose A. Salinas-Perez, Maria Luisa Rodero-Cosano, Sonia Conejo-Cerón

**Affiliations:** 1 Department of Psychology, Universidad Loyola Andalucía, 41704 Sevilla, Spain; 2 Prevention and Health Promotion Research Network (redIAPP), 08007 Barcelona, Spain; 3 Department of Quantitative Methods, Universidad Loyola Andalucía, 41704 Sevilla, Spain; 4 Centre for Mental Health Research, Australian National University, Canberra, ACT 2601, Australia; 5 Biomedical Research Institute of Malaga (IBIMA), 29009 Málaga, Spain

**Keywords:** social determinants, mental health, socioeconomic factors, health services, risk factors, protective factors

## Abstract

Mental disorders are one of the greatest public health concerns of our time, and they are affected by social factors. To reduce the considerable burden of mental disorders, more global and systematic knowledge of the social determinants of mental health is necessary. This paper presents the results of the 27 studies included in the *International Journal of Environmental Research and Public Health* Special Issue, “Social Determinants of Mental Health”. The studies are grouped into four broad categories: social inclusion and mental health, young people’s mental health, mental health at work, and mental health service users. The results cover different countries, age populations, settings, and methodologies. Finally, the main findings on the relationship between social determinants and mental health are presented and summarized.

## 1. Introduction

Mental disorders are one of the greatest public health concerns of our time. Mental disorders have consistently accounted for more than 14% of years lived with disability for nearly three decades, and they have a greater than 10% prevalence in all 21 Global Burden of Diseases regions [1]. Depression and anxiety are the most prevalent mental disorders [2]. Currently, depression ranks third (women) and fifth (men) in global disease burden [1], and it is expected to be the first in developed countries by 2030 [3]. The considerable burden of mental disorders, in both sexes and across all age groups, substantiates a global need for increased mental health research [1].

There is strong evidence that mental disorders are influenced by social determinants [4]. The social determinants of mental health are social and economic factors (such as socioeconomic status, education, neighbourhood, employment, social support networks, and health services) that influence people’s mental health [5]. These social factors are strongly associated with social inequalities, whereby the greater the social inequality, the higher the risk [6]. Therefore, more global and systematic knowledge of social determinants of mental health is needed [7].

The goal of this editorial is to present a broad review of the results of the international studies that have been included in the *IJERPH* Special Issue, “Social Determinants of Mental Health,” to provide the latest evidence in this field. This special issue was intended to make a substantial contribution to knowledge gaps in understanding how social determinants influence mental health in many positive and negative ways. A wide range of topics were included related, but not limited, to: stress, living conditions, education, unemployment and job security, employment and working conditions, housing, social exclusion, mental health services, gender, ethnicity, and disability. This Special Issue has become a new *IJERPH* Topical Collection within the Mental Health section.

## 2. Methods

All of the studies accepted for publication in the *IJERPH* Special Issue “Social Determinants of Mental Health” were included in this review. The submission call was open from October 2019 to August 2020.

All studies were reviewed in full text, and their principal characteristics (authorships and year of publication, country, target population, methodology, outcomes and main findings) were extracted in an evidence table. All of the review and summary processes were carried out by the four editors of the Special Issue.

## 3. Results

The issue finally included 27 studies. Table 1 shows a synthesis of all articles accepted for publication in the Special Issue, “Social Determinants of Mental Health”.

The methods of the accepted publications included quantitative (*N* = 21), qualitative (*N* = 4), and review methods (*N* = 2). The studies were based on samples from countries of Asia and Oceania (*N* = 11), Europe (*N* = 7), North America (*N* = 3), and Central and South America (*N* = 3). In addition, three studies had an international focus and analysed data from different countries. The studies addressed a wide variety of topics, although they could be grouped into four broad categories: social inclusion and mental health, young people’s mental health, mental health at work, and mental health service users.

### 3.1. Social Inclusion and Mental Health

People with mental disorders are one of the most marginalized groups in our societies, and they face discrimination and stigma [8]. The special issue addressed this question through eight articles that focused on the mediating role of social inclusion in mental health.

One of them studied the influence of isolation and rural adversity on mental health, describing a conceptual framework for this problem [9]. In the second one [10], the researchers analysed how the characteristics of the neighbourhood can influence poor mental health and how it is necessary to take them into account to develop national policies and programmes that influence and improve the health of residents. Another article analysed protective and risk factors for the mental health of Rohingya refugees [11]. This study concluded that violence, food insecurity, and low social support significantly increase the odds of mental health diseases, and more efforts are needed to address these issues. Another piece of research studied the mediating effect that cognitive and structural social capital has on the influence of education on depression and obesity and how it is possible to use this effect to improve habits and education about healthy habits, which will improve both obesity and depression, among elderly people [12]. A fifth article studied the effect of religiosity on mental health, concluding that attachment and closeness to God seem important for improving mental health, but it is necessary to consider other socioeconomic factors. In addition, this article considered that a unification of the indicators that measure religiosity is necessary since the results vary widely according to the selected indicators [13]. Other research has demonstrated that the hedonic and utilitarian performances of volunteer tourism significantly and positively contribute to increasing travellers’ mental health, which ultimately enhances their prosocial intentions [14]. Last, community integration of persons with mental disorders was another topic addressed in a Korean sample [15]. The comparison with the general population showed differences in the effects of sociodemographic variables on social integration between the groups, so social networks and social contacts were both reduced among persons with mental disorders.

### 3.2. Young People’s Mental Health

A total of seven articles in the Special Issue were conducted on young people, including children, adolescents, and young adults. The studies addressed two relevant topics for young people’s mental health: mental well-being, an essential issue for preventing mental disorders in adulthood [36], and suicidality, currently a leading cause of death of young people worldwide [37].

Five articles discussed mental well-being among young people. The first article estimated the prevalence of depression, anxiety, and stress in a cross-sectional study of 1074 Spanish college students [31]. They found a significant prevalence of symptoms of stress (34.5%), anxiety (23.6%), and depression (18.4%) in their population. A second article analysed the association between neurocognitive domains and subjective well-being [29]. The results revealed that subjective well-being was associated with different neurocognitive domains (e.g., executive control, episodic memory, complex cognition, and social cognition) in a sample of Spanish adolescents. A third article aimed to understand the relationship between control and response to stress in the prediction of mental well-being in university and undergraduate students in Hong Kong [35]. The results showed that while external control was positively related to avoidant coping, fate control was positively related to both active and avoidant coping. Another article addressed the relationship between risky behaviours (e.g., drug use or unsafe sex) and mental health in Brazilian students aged 11 to 19 years [19]. The authors found that students with mental health symptoms were more frequently involved in risky behaviours. The last article analysed the relationships that sociodemographic and socioeconomic factors had with the psychological adjustment of low socioeconomic status (SES) Guatemalan children and adolescents and how these relationships were mediated by food insecurity and exposure to violence [18]. The results revealed that violence exposure was positively related to both depression and anxiety and negatively related to health-related quality of life. Food insecurity did not seem to influence the psychological adjustment outcomes in this low-SES sample.

Suicide among adolescents was addressed by two articles. The first article designed a cross-sectional study to analyse the association between demographic and socioeconomic factors and depressive symptoms and suicidal ideation in referred and non-referred adolescents [16]. Referred adolescents showed higher levels of depressive symptoms and suicidal ideation than non-referred adolescents. The results of moderation analyses showed that age, in referred adolescents, and SES, in non-referred adolescents, moderated the relationship between depressive symptoms and suicidal ideation. The second article aimed to understand the living conditions of Mexican adolescents who had attempted suicide through a qualitative study [34]. The findings pointed out that poverty rendered the daily lives of adolescents precarious, compromising even their basic needs.

### 3.3. Mental Health at Work

The burden of work-related mental health is enormous. Mental illness is now the leading cause of both sickness absence and incapacity benefits in most high-income countries, accounting for 40% of certified sicknesses in primary care [38]. Furthermore, unemployment is considered a recognized risk factor for common mental disorders, such as depression and anxiety [39]. The special issue included six articles focused on work-related determinants of mental disorders in different populations.

A review of 45 studies focused on work-related psychosocial stress among employees of small and medium-sized enterprises [32]. They found that the type of the studies and the themes analysed were heterogeneous, and more studies with observational or experimental designs are needed. Moreover, work-related psychosocial factors were studied in primary care teams in another article [33]. The results of the qualitative study showed that psychological factors related to the work content and tasks, the organization of the work, and the working environment were the principal demands of the general practitioners interviewed in Germany. A third study was focused on the needs of migrant workers in China [26]. They are an important human resource for the economic and social development of the country, and their conditions of life could be improved. The findings of the study pointed out that age, educational background, contracted land, collective dividends, and income were factors related to happiness. The conversion of household registration could improve the happiness of migrant workers with low educational backgrounds, low income, and contracted land. Another piece of research studied how a threatening event, such as the Great East Japan earthquake that occurred in 2011, impacted the affected population [25]. This disaster caused a high rate of unemployment in the coastal communities, which in turn negatively impacted their mental health. The results highlighted that the impact was even higher among workers in the primary industry, women, and elderly populations. Another study examined the factors that impact job retention among people with schizophrenia in Korea [22]. They observed significant differences between participants who did and did not hold a job for six months or more in terms of the clinical characteristics of psychotic symptoms, global functioning, and interpersonal functioning; the vocational characteristics of the type of employment, income, and work hours; theory of mind as related to social cognition; and hostility and blame attribution perceptions. Finally, the last paper analysed how the employment situation can influence the mental health of Korean employees [30], concluding that precariousness and temporary employment affect mental health more than stable employment. Additionally, stressed women have an increased habit of smoking. However, higher alcohol consumption was associated with men with stable jobs as a means of socialization.

### 3.4. Mental Health Service Users

The characteristics of the mental services, such as availability, access, type of care provided and funding, have a relevant influence on the population’s mental health status along with contextual factors, such as national incomes and individual socioeconomic factors [40]. Social determinants in people with mental disorders were studied in seven articles.

Three articles aimed to study the relationships between mental health care and patient sociodemographic, clinical, and self-perception factors. Their findings pointed out an association between the use of services and health self-perception in a Spanish study [21] and the influence of having social and severe clinical problems on being a beneficiary of a specific recovery intervention based on one’s personal budget in Italy [20]. The last article analysed the wide variation by ethnicity in the prevalence, course, and unmet treatment needs of their psychiatric disorders. Additionally, it focused on the need to attend to differences in this diversity to target prevention efforts and mental health service planning about the specific needs of these black subpopulations in the USA [24].

Another article studied the effect of national income on population mental health in 55 countries [23]. The impact of national income was heterogeneous for different mental disorders and countries with socioeconomic variables as moderating factors.

Finally, three studies focused on three specific issues for people with mental disorders: stigma, suicidality, and drug misuse. The former study focused on developing an instrument to assess self-stigma among partners of persons with autism spectrum disorder in Japan, thus contributing to the self-stigma theory in the case of mental health patient partners [28]. The second study carried out a systematic review on suicide risk with a meta-analysis focused on the European population [17]. In the general European population, several factors have a significant association with nonlethal suicidality with the highest correlation for clinical factors, followed by psychosocial factors and, finally, demographic factors. This research also overcame some difficulties in comparing these kinds of studies. Finally, the latter study aimed to identify opioid misuse risk profiles in the USA [27]. By using a person-centred approach, this study described five helpful profiles, considering sociodemographic and health indicators and substance use, to develop targeted interventions to prevent substance use disorders and their adverse consequences.

## 4. Discussion

This paper provided an overview of the 27 studies submitted to the *IJERPH* Special Issue, “Social Determinants for Mental Health”. The results are focused on social determinants divided into four main categories: social inclusion and mental health, young people’s mental health, mental health at work, and mental health service users. The results highlight the importance of increasing the evidence of how and why social, economic, and physical environments in which people live influence the mental health of the population. The evidence-based results can boost actions for preventing mental disorders and promote mental health across society.

Social determinants can be mediators (how or why a particular effect or relationship occurs) or moderators (variables that change the strength of an effect or relationship between two variables) with significant results for mental health. A group of articles analysed social inclusion variables, including isolation and rural adversity, characteristics of the neighbourhood, factors related to refugees, social capital, employment, psychosocial stress, personality characteristics, and religiosity. Another group of studies confirmed that mental disorders in adolescents and students are currently associated, in addition to psychological factors, with social factors, such as poverty or violence exposure. Moreover, the results show that working conditions have a powerful influence on the well-being of workers. Psychosocial stress, workers’ needs and demands, and unemployment have also been identified as work-related determinants of mental health. Last, health self-perception and SES are other factors that can influence mental health status, and mental health status influences the use of mental health services.

The main limitation of this special issue is that the studies included are mostly cross-sectional and focused on adults. More studies with longitudinal and experimental designs are needed. Additionally, more studies that include other stages of the life course, such as pregnancy, childhood, and ageing, are needed. There is also a distinct lack of studies testing preventive interventions for common mental disorders that focus on the main social determinants of the entire population.

## 5. Conclusions

The articles submitted to the *IJERPH* Special Issue, “Social Determinants for Mental Health,” include a wide range of studies addressing the relationships between social determinants and mental health from different research approaches and methods. This Special Issue received outstanding research studies coming from all over the world, which indicates its current interest and increasing relevance. The editors hope that the *IJERPH* Topical Collection will bring about significant advances in this field of research in the coming years.

## Figures and Tables

**Table 1:** Description of the articles included in the Special Issue in alphabetical order.

Authorships Year	Country	Target Population	Methodology	Outcomes	Main Findings
Antolín-Suárez et al., 2020 [16]	Spain	Outpatient adolescents using mental health facilities (referred group) and community adolescents (non-referred group)	Quantitative	Depressive symptoms and suicidal ideation. Moderating role of demographic, social, and economic factors	Results showed higher levels of depressive symptoms and suicidal ideation in the referred group. The moderation analyses showed that age, in referred adolescents, and socioeconomic status, in non-referred adolescents, moderated the relationship between depressive symptoms and suicidal ideation
Carrasco-Barrios et al., 2020 [17]	Different European countries^*^	European general population	Systematic review and Meta-analysis	Suicidality, death wishes, suicidal plans, suicidal ideation, suicidal attempts and risk factors	The general European population shows that several factors have a significant association with non-lethal suicidality, with the highest for clinical factors, followed by psychosocial factors and, finally, demographic factors. This research improves on some difficulties to compare these types of studies
Company-Córdoba et al., 2020 [18]	Guatemala	Low-socioeconomic status children and adolescents	Quantitative	Perceived quality of life, depression and anxiety	Exposure to violence significantly moderates the effect of sociodemographic and socioeconomic variables on measures of depression, anxiety and health-related quality of life
Escobar et al., 2020 [19]	Brazil	Students from schools involved in risky behaviours	Quantitative	Risky behaviours and mental health	Students with symptoms of mental health issues were involved in risky behaviours, including drug use and unsafe sex
Fontecedro et al., 2020 [20]	Italy	Individual Health Budgets (IHB) in mental health	Quantitative	Socioeconomic and clinical characteristics, type of IHB	Identification of clinical and socioeconomic features of people with mental disorders benefitting from an IHB intervention
Forthman et al., 2021 [10]	U.S.A.	Neighbourhood from U.S.A.	Quantitative	Neighbourhood Mental Health	The findings show that neighbourhood characteristics are strongly related to mental health, indicating the importance of the factor model in future research focused on the influence of neighbourhood characteristics on mental health. Policymakers and public health professionals can use this information to better understand how (1) the characteristics of their communities and (2) policies and programmes (including those that may not appear directly related to health) have significant impacts on the health of its residents
González-Suñer et al., 2021 [21]	Spain	Patients with major depressive disorder in primary care setting	Quantitative	Use of mental health services	Having previously used mental health services was associated with the use of mental health services. The use of public mental health services was associated with a worse perception of quality of life. No other sociodemographic, clinical, nor FP variables were associated with the use of mental health services
Han and Jun, 2020 [22]	Korea	Patients with schizophrenia	Quantitative	Psychotic symptoms, interpersonal functioning, social cognition	Theory of mind, attribution style, and psychotic symptoms explained 52.7% of the variance in job retention
Han et al., 2020 [14]	China	Volunteer tourists	Quantitative	Prosocial intention, hedonic performance, utilitarian performance, mental health, engagement, problem awareness, ascribed responsibility	The volunteer tourism engagement and prosocial intention relation was under the significant influence of problem awareness and ascribed responsibility
Huang et al., 2020 [23]	55 different countries around the world	Impacts of national incomes on mental health	Quantitative	National income, socioeconomic factors, anxiety and depression prevalence	Heterogeneous impact of national incomes on different types of mental health and countries
Jones et al., 2020 [24]	U.S.A.	African American, U.S. and foreign-born Caribbean women	Quantitative	Mental health, ethnicity, nativity, psychopathology	Despite a lower prevalence of psychiatric disorders in black women, there is a great likelihood their disorders will be marked by persistence, which underscores the need for culturally specific treatment approaches
Katayanagi et al., 2020 [25]	Japan	Adults	Quantitative	Mental health, employment status	The Great East Japan Earthquake negatively impacted employment and the mental health of the affected population
Kaur et al., 2020 [11]	Malaysia	Rohingya refugees	Quantitative	Mental health disorders	The most common mental health disorders affecting Rohingya refuges were generalized anxiety disorders, followed by posttraumatic stress disorders and major depressive disorders. Factors such as low social support, food insecurity, exposure to violence, and duration since displacement were found to be risk factors for developing mental health disorders among this population
Lawrence-Bourne et al., 2020 [9]	Australia	Rural population	Qualitative	Rural adversity (disasters, rural epidemiology, theories and frameworks of rurality, rural risk and protective factors, and the impact of rural adversity on mental health)	Rural adversity can be understood using a rural ecosystem lens to develop greater clarity around the dimensions and experiences of adversity and to help identify the opportunities for interventions
Lee and Seo, 2020 [15]	Korea	General population	Quantitative	Physical integration, social integration, psychological integration, social network size, social contact frequency and community integration	The effects of socio-demographic variables on the three types of community integration differed between the two groups. Persons with mental disorders had smaller social networks and fewer contacts than the general population
Liu et al., 2020 [26]	China	Migrant workers	Quantitative	Household registration, happiness	Age, educational background, contracted land, collective dividends, and income significantly affect the improvement of happiness
Malinakova et al., 2020 [13]	Czech Republic	Czech population	Quantitative	Religiosity, anxiety in close relationships and other mental health problems	The heterogeneity of findings in associations between religiosity/spirituality and mental health could be due to measurement problems and variations in the degree of secularity
Montiel et al., 2020 [27]	U.S.A.	Noninstitutional adults reporting opioid misuse	Quantitative	Opioid, analgesics, latent class analysis, social determinants of health	Five misuse profiles were identified
Naoko et al., 2020 [28]	Japan	Partners of persons with Autism Spectrum Disorder	Qualitative	Autism Spectrum Disorder, couple stigma, self-stigma, construct validity	The Japanese version of the Couples Stigma Scale is a valid instrument for assessing self-stigma in the spouses of persons with Autism Spectrum Disorder
Ortuño-Sierra et al., 2020 [29]	Spain	Adolescents at risk for low personal well-being	Quantitative	Neurocognitive domains and subjective well-being	Adolescents with low personal well-being showed statistically significant impairments across the different neurocognitive domains. Adolescents at risk showed lower accuracy scores on executive function and complex cognition and lower speed scores on episodic memory, complex cognition, and social cognition scores
Park et al., 2020 [30]	South Korea	South Korean employees	Quantitative	Mental health, health behaviour, general health and employment status	Temporary workers and the unemployed have higher odds of poor mental health regardless of gender. Male permanent workers were found to have a higher risk of problematic drinking. Women with temporary jobs had a higher risk of current smoking
Ramón-Arbués et al., 2020 [31]	Spain	College students	Quantitative	Depression, anxiety, stress symptoms	Moderate prevalence of depression (18.4%), anxiety (23.6%), and stress (34.5%) symptoms
Schreibauer et al., 2020 [32]	Different countries around the world	Workforce in small and medium-sized enterprises	Integrative review	Health or health-related outcomes (general, work-related), stress outcomes, health, well-being, factors affecting cardiovascular health, mental health, musculoskeletal system, social relations, and business-related outcomes)	This review underlines the need for more and better-quality research on psychosocial factors in small and medium-sized enterprises
Tsarouha et al., 2020 [33]	Germany	General practitioners (GPs)	Qualitative	Psychosocial demands	General practitioners’ psychosocial demands included factors related to work content and tasks, organization of work, and the working environment
Valdez-Santiago et al., 2020 [34]	Mexico	Adolescents who attempted suicide	Qualitative	Understanding suicidal behaviour	Poverty, manifested primarily as material deprivation, rendered the daily lives of adolescents precarious, compromising even their basic needs. All of the young people analysed had either received medical, psychological, and/or psychiatric care as outpatients or had been hospitalized. School played a positive role in referring adolescents with suicidal behaviour to health services; however, it also represented a high-risk environment
Wu et al., 2020 [35]	China	Undergraduate and university students	Quantitative	Fate control, coping strategies, locus of control	External control was positively related to avoidant coping; fate control was positively related to both active and avoidant coping
Xin and Ren, 2020 [12]	China	Population of China	Quantitative	Education, depression, obesity, and structural social capital and cognitive social capital	Social capital as a mediator through the effect of education on depression and obesity among the elderly in China. It is possible to use social capital (cognitive social capital and structural social capital) to adjust its relationship with health among the elderly in China
